# Nerve growth factor and receptor expression in rheumatoid arthritis and spondyloarthritis

**DOI:** 10.1186/ar2716

**Published:** 2009-06-02

**Authors:** Christian Barthel, Nataliya Yeremenko, Roland Jacobs, Reinhold E Schmidt, Michael Bernateck, Henning Zeidler, Paul-Peter Tak, Dominique Baeten, Markus Rihl

**Affiliations:** 1 Clinic for Immunology and Rheumatology, Hannover Medical School (MHH), Carl-Neuberg-Strasse 1, Hannover 30625, Germany; 2 Division of Clinical Immunology and Rheumatology, Academic Medical Center (AMC), University of Amsterdam, Meibergdreef 9, Amsterdam, 1105, The Netherlands; 3 Department of of Anesthesiology, Pain Clinic, Hannover Medical School (MHH), Carl-Neuberg-Strasse 1, Hannover, 30625, Germany; 4 Rheumatologikum Hannover, Rathenaustrasse 13/14, Hannover, 30159, Germany

## Abstract

**Introduction:**

We previously described the presence of nerve growth factor receptors in the inflamed synovial compartment. Here we investigated the presence of the corresponding nerve growth factors, with special focus on nerve growth factor (NGF).

**Methods:**

mRNA expression levels of four ligands (NGF, brain derived growth factor (BDNF), neurotrophin (NT)-3, NT-4) and their four corresponding receptors (tyrosine kinase (trk) A, trkB, trkC, NGFRp75) were determined in the synovial fluid (SF) cells of 9 patients with rheumatoid arthritis (RA) and 16 with spondyloarthritis (SpA) and compared with 7 osteoarthritis (OA) patients. NGF was also determined in synovial tissue (ST) biopsies of 10 RA and 10 SpA patients. The production of NGF by monocytes and lymphocytes was assessed by flow cytometry of SF cells, synovial tissue derived fibroblast-like synoviocytes (FLS) were assessed by ELISA on culture supernatant.

**Results:**

SF cell analysis revealed a clear BDNF and NGF mRNA expression, with significantly higher NGF expression in RA and SpA patients than in the OA group. NGF expression was higher in ST samples of RA as compared to SpA. Using intracellular FACS analysis, we could demonstrate the presence of the NGF protein in the two inflammatory arthritis groups on both CD3+ T lymphocytes and CD14+ cells, i.e. monocytes/macrophages, whereas cultured FLS did not produce NGF *in vitro*.

**Conclusions:**

Neurotrophins and especially NGF are expressed in the synovial fluid and tissue of patients with peripheral synovitis. The presence of neurotrophins as well as their receptors, in particular the NGF/trkA-p75 axis in peripheral synovitis warrants further functional investigation of their active involvement in chronic inflammatory arthritis.

## Introduction

There is increasing evidence for the presence of neuronal growth factors in chronic inflammatory arthritis. Neurotrophins (nerve growth factor (NGF), brain-derived neurotrophic factor (BDNF), and the neurotrophins NT-3 and NT-4) constitute a family of growth factors essential for the development, proliferation, differentiation, and survival of neuronal as well as various non-neuronal cells. Neurotrophins bind to their specific high-affinity receptors tyrosine kinase (trk) A (NGF), trkB (BDNF, NT-4), and trkC (NT-3), and to one low-affinity receptor p75 (or NGFRp75) that binds to all ligands. This p75 receptor is a member of the TNF receptor superfamily [1]. In particular, the NGF-trkA/p75 axis arouses increasing interest due to its role in chronic inflammatory arthritis in which a pathogenic function of this system has been postulated [2-6].

Using immunohistochemistry, we previously performed a detailed analysis on the synovial expression of neurotrophins showing convincingly high levels of both the trkA and the p75 NGF receptors in peripheral synovitis of patients with spondyloarthritis (SpA). Their expression correlated with signs of inflammation and was modulated by effective treatment with anti-TNF [7]. However, apart from BDNF we were unable to demonstrate the presence of the ligands at the protein level. Accordingly, this study was designed to assess the expression of NGF and all other known neurotrophic ligands (BDNF, NT-3, NT-4), as well as their receptors (trkA, NGFRp75, trkB, trkC), in order to provide evidence that all actors of this system are present and actively upregulated in the inflamed synovial compartment.

## Materials and methods

### Patients

Synovial fluid (SF) samples were collected from nine patients with rheumatoid arthritis (RA) fulfilling the American College of Rheumatology classification criteria and from 16 patients with SpA fulfilling the European Spondyloarthropathy Study Group classification criteria [8,9]. Also, seven patients with osteoarthritis (OA) served as non-inflammatory controls. All patients had active synovitis of the knee. Patient characteristics and disease activity parameters are listed in Table 1. OA patients were graded according to the Kellgren and Lawrence classification [10]. The majority of patients had OA of the knee grade 2 (mean 2.3 ± standard deviation (SD) 1.0).

**Table 1:** Clinical characteristics of neurotrophins and receptors in the synovial fluids of SpA, RA, and OA patients

**Patient Groups**		**Clinical characteristics**								
		
		**age**	**gender**	**DD (y)**	**SJC**	**TJC**	**CRP (mg/l)**	**ESR (mm)**	**SFc/μl**	**%PMN**
**SpA (n = 16)**	median	**37**	6 f/10 m	**5**	2*	1*	10.5*	**20**	7025*	**72**
	SD	16.8		5.9	1.2	1.9	23	15	8825	26
	(range)	(16 to 65)		(0.3 to 18)	(1 to 4)	(0 to 8)	(1 to 99)	(3 to 54)	(2500 to 31100)	(5 to 92)

**RA (n = 9)**	median	**49**	8 f/1 m	**5**	**5#**	**3#**	**11.7#**	**26**	**7950#**	**76**
	SD	15.3		9.2	3.2	1.8	29	8.8	3645	11
	(range)	(37 to 77)		(1 to 26)	(2 to 13)	(2 to 7)	(7.5 to -95)	(12 to 67)	(2600 to 1300)	(50 to 90)

**OA (n = 7)**	median	**67**	5 f/2 m	**6**	**1**	**1**	**3**	**17**	**700**	**60**
	SD	5.8		5.7	0.5	0.4	1.1	5.6	1096	33
	(range)	(58 to 75)		(2 to 18)	(1 to 2)	(1 to 2)	(1.6 to 4.5)	(12 to 25)	(100 to 3250)	(5 to 90)

Table 1 shows the clinical data and parameters of disease activity of 16 spondyloarthritis (SpA), 9 rheumatoid arthritis (RA), and 7 osteoarthritis (OA) patients used for the RT-PCR measurements on the synovial fluid samples. Swollen joint count (SJC), tender joint count (TJC), C-reactive protein (CRP), and synovial fluid (SF) leukocyte counts were higher in both SpA (*) and RA (^#^) as compared with OA; Mann Whitney U test; *P *< 0.05).

DD = disease duration given in years; ESR = erythrocyte sedimentation rate; f = female; m = male; PMN = percentage of polymorphonuclear cells of SF leukocytes; OA = osteoarthritis; RA = rheumatoid arthritis; SD = standard deviation; SpA = spondyloarthritis.

Synovial tissue (ST) biopsies were obtained from another panel of patients including 10 SpA and 10 RA patients with early and active arthritis of the knee (disease duration <6 months, only NSAID treatment, but no steroids, no disease-modifying anti-rheumatic drugs, or biologics).

All subjects gave their written informed consent before inclusion in the study, which was approved by the local ethics committee of the involved institutions.

### Synovial fluid and synovial tissue biopsy samples and extraction of total RNA

SF was obtained by a conventional puncture of an actively inflamed knee joint. Samples were centrifuged for 15 minutes at 1000 g. Supernatants were removed, whole SF cell pellets were resuspended in RNAlater solution (Ambion, Austin, TX, USA), and frozen at -80°C until use. For intracellular fluorescence-activated cell sorting (FACS) analysis, mononuclear cells derived from SF samples (SFMC) were obtained by standard Ficoll histopaque procedure and conserved in FCS and 10% dimethyl sulfoxide. They were kept in liquid nitrogen until use. ST biopsies were obtained by a standard procedure as previously described [11]. The Ficoll procedure was also used in order to obtain peripheral blood mononuclear cells (PBMC) from four healthy individuals used as controls for PCR. Total RNA was extracted from SF cell pellets, ST biopsies, and PBMC using TRizol reagent (Invitrogen, Karlsruhe, Germany) and precipitation with isopropyl alcohol. All procedures were previously described in detail [12-14].

### Quantitative real-time RT-PCR (TaqMan assay)

Generation of cDNA by reverse transcription and utilization of TaqMan^® ^assay followed the protocol of the manufacturer (Applied Biosystems, Darmstadt, Germany). Briefly, 2 μg of total RNA were reverse transcribed using the MultiScribe^® ^(Applied Biosystems, Darmstadt, Germany) reverse transcriptase. Duplicate PCR reactions were performed using the TaqMan^® ^universal PCR master mix on ABI Prism^® ^7000 sequence detection system (Applied Biosystems, Darmstadt, Germany). After denaturation at 50°C for two minutes and 95°C for 10 minutes, 45 PCR reaction cycles were performed each at 95°C for 9 seconds and 60°C for one minute. The following mRNA transcripts and assays were used (Applied Biosystems, Warrington, UK): assays-on-demand for NGF-beta (assay no. Hs00171458), [GenBank:X52599], BDNF (assay no. Hs00156058), [GenBank:M61176], and NT-3 (assay no. Hs00267375), [GenBank:M37763]; assay-by-design for NT-4 (cat. no. 4332078; [GenBank:M86528]); assays-on demand for the high-affinity receptors trkA (assay no. Hs00176787), [GenBank:X03541]; trkB (assay no. Hs00178811), [GenBank:U12140]; trkC (assay no. Hs00176797), [GenBank:U05012], and for the low-affinity receptor NGFRp75 (assay no. Hs00609976), [GenBank:M14764].

### Delta delta Ct method and statistical analysis

All PCR data were normalized to the expression of the glyceraldehyde 3-phosphate dehydrogenase (G3PDH) housekeeping gene used as an internal control; SF data were also normalized with a set of four healthy PBMC used as an external control. The ST data were compared with each other. PCR data were obtained as cycle threshold (Ct) values. The Ct value is defined as the cycle with a fluorescence intensity significantly above the background fluorescence but within the exponential phase of the amplification [15,16]. The mean of two Ct measurements of one sample was calculated for both the given target gene and the G3PDH gene. Delta Ct was determined as the mean of the duplicate Ct values for the target gene subtracted by the mean of the duplicate Ct values for the G3PDH gene. For each target gene, delta Ct measurements were performed separately for both the SF samples and the healthy PBMC. The delta delta Ct method represents the difference between the two cell types for a given target gene [17]. Expression levels are given as fold of expression and were compared between SpA, RA, and OA groups using the non-parametric Mann Whitney U test as appropriate.

### Fluorescence-activated cell sorting for detection of NGF

SF mononuclear cells (SFMC) of three RA and three SpA patients from the panel described in Table 1, as well as PBMC from two healthy controls were prepared by density gradient centrifugation using biocoll (1.077 g/ml; Biochrom, Berlin, Germany). SFMC were harvested from the interphase and washed twice at 1000 g and 300 g, respectively. The cells were finally resuspended in PBS/BSA and stained for surface markers (CD3 PerCP, clone SK7; CD14 FITC, Leu-M3; CD56 APC, NCAM 16.2; all from Becton Dickinson, Heidelberg, Germany) for 20 minutes. After two washes with PBS/BSA (300 g/three minutes) the cells were fixed for 10 minutes at room temperature in PBS containing 4% paraformaldehyde. Cells were then washed once and resuspended in saponin buffer (PBS supplemented with 5 mM HEPES and 0.1% saponin) in order to perforate the cell membranes. Subsequently, aliquots were stained with monoclonal antibodies (mAb) against NGF (biotinylated anti-human β-NGF, catalogue number BAF256; R&D systems, Minneapolis, MN, USA). Unspecific binding of the mAb via Fc-receptors was discriminated by adding human IgG solution (Octagam; Octapharma, Langenfeld, Germany). After 30 minutes of incubation at 4°C, cells were washed three times with PBS/BSA and resuspended again in saponin buffer. PE-labeled streptavidin (SA-PE, Becton Dickinson, Heidelberg, Germany) for secondary staining of biotinylated NGF was added and cells were incubated for 30 minutes at 4°C. After three washes (300 g/three minutes) with PBS/BSA, cells were ready for FACS analysis.

Phenotypic analyses were performed as multicolor immunofluorescences. At least 10^4 ^cells per appropriate lymphocyte or monocyte gate, respectively, according to forward scatter vs side scatter properties were analyzed using a dual-laser cytometer with Cell Quest Pro (FACSCalibur, Becton Dickinson, Heidelberg, Germany) and Summit 4.3 (Beckman Coulter, Krefeld, Germany) software.

### Culture of fibroblast-like synoviocytes to determine NGF production

Fibroblast-like synoviocytes (FLS) were isolated from synovial biopsies of one RA and one SpA patient as described previously [18]. After three passages, cells were resuspended at 10,000 cells/ml Dulbecco's modified eagle's medium (DMEM) with 10% FCS and plated at 2 ml/well in 24 well plates. Cells were grown for three days until confluence, then the normal medium (DMEM + 10% FCS) was replaced by starvation medium (DMEM + 1% FCS). After 24 hours, cells were stimulated with either medium alone (DMEM 1% FCS); TNF-alpha at 10 ng/ml; or IL-1 beta at 10 ng/ml, or lipopolysaccharide at 1 ug/ml. After 72 hours of culture with stimulation, supernatants were collected and used undiluted for measuring NGF by ELISA (R&D Systems, Minneapolis, MN, USA; lowest detection level: 30 pg/ml).

## Results

**Figure 1:**
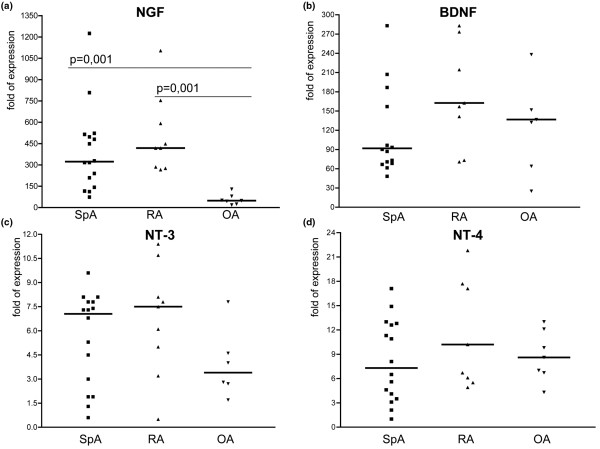
Scatter plots showing mRNA expression of the neurotrophic ligands. The scatter plots a to d depict the expression levels of the four neurotrophic ligands. **(a) **nerve growth factor (NGF). **(b) **Brain-derived growth factor (BDNF). **(c) **Neurotrophin (NT)-3. **(d) **NT-4). Bold horizontal lines represent the median. The highest levels were found for BDNF and NGF. Significantly higher expression was revealed for NGF in both spondyloarthritis (SpA) and rheumatoid arthritis (RA) as compared with osteoarthritis (OA; *P *= 0.001 for both comparisons).

**Figure 2:**
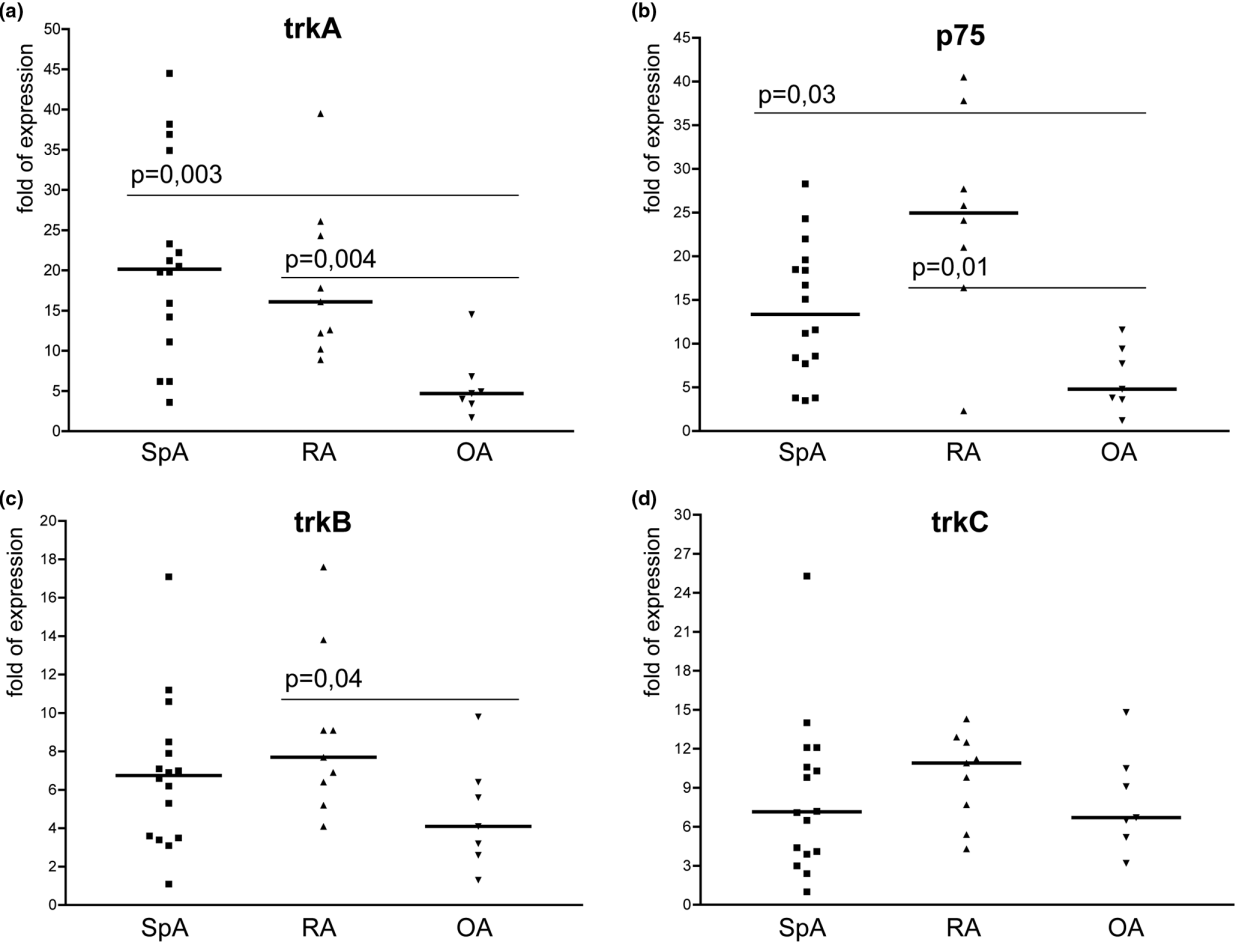
Scatter plots showing mRNA expression of neurotrophin receptors. The scatter plots a to d depict the expression levels of the four neurotrophin receptors. **(a) **Tyrosine kinase (trk)A. **(b) **p75. **(c) **trkB. **(d) **trkC. Bold horizontal lines represent the median. The highest levels were found for trkA and p75, revealing significantly higher expression levels in spondyloarthritis (SpA; *P *= 0.0003, *P *= 0.003 respectively) and rheumatoid arthritis (RA; *P *= 0.004 and *P *= 0.001, respectively) vs osteoarthritis (OA).

The mRNA expression levels of the four neurotrophic ligands and the four receptors as determined in the SF cells are depicted in Table 2 and outlined in detail by scatter plots in Figure 1 (ligands) and Figure 2 (receptors).

**Table 2:** RT-PCR expression levels of neurotrophins and receptors in the synovial fluids of SpA, RA, and OA patients

**Patient Groups**		**RT-PCR results**								
		
		**TrkA**	**p75**	**trkB**	**trkC**	**NGF**	**BDNF**	**NT-3**	**NT-4**	
**SpA (n = 16)**	median	**20**	**13.4**	**6.7**	**7.1**	**323**	**92**	**7.0**	**7.3**	
	SD	12	7.7	3.9	6.0	296	113	2.9	5.0	
	(range)	(3.6 to 45)	(3.5 to 28)	(1.1 to 17)	(2.4 to 25)	(74 to 1225)	(71 to 444)	(1.3 to 9.6)	(1 to 15)	

**RA (n = 9)**	median	**16**	**25**	**7.7**	**11**	**418**	**163**	**7.5**	**10**	
	SD	10	12	4.3	3.4	275	101	3.5	7 to 6	
	(range)	(8.9 to 40)	(2.3 to 41)	(4.1 to 18)	(4.3 to 14)	(267 to 1104)	(71 to 374)	(0.5 to 11)	(4.9 to 22)	

**OA (n = 7)**	median	**4.7**	**4.8**	**4.1**	**6.7**	**49**	**137**	**3.4**	**8.6**	
	SD	4.2	3.7	2.8	3.8	37.7	98.5	2.2	3.1	
	(range)	(1.7 to 15)	(3.2 to 15)	(1.3 to 9.8)	(3.2 to 15)	(18 to 129)	(25 to 238)	(1.7 to 7.8)	(4.3 to 13)	

Table 2 shows the RT-PCR expression levels of mRNA transcripts of four neurotrophins (nerve growth factor (NGF), brain-derived growth factor (BDNF), and neurotrophin (NT)-3, NT-4) and their corresponding receptors (high-affinity receptors tyrosine kinase (trk)A, trkB, trkC and the low-affinity NGF receptor p75) of all 32 patients are shown (see also Figures 1 and 2 for detailed data and statistics).

DD = disease duration given in years; ESR = erythrocyte sedimentation rate; f = female; m = male; PMN = percentage of polymorphonuclear cells of SF leukocytes; OA = osteoarthritis; RA = rheumatoid arthritis; SD = standard deviation; SpA = spondyloarthritis.

**Figure 3:**
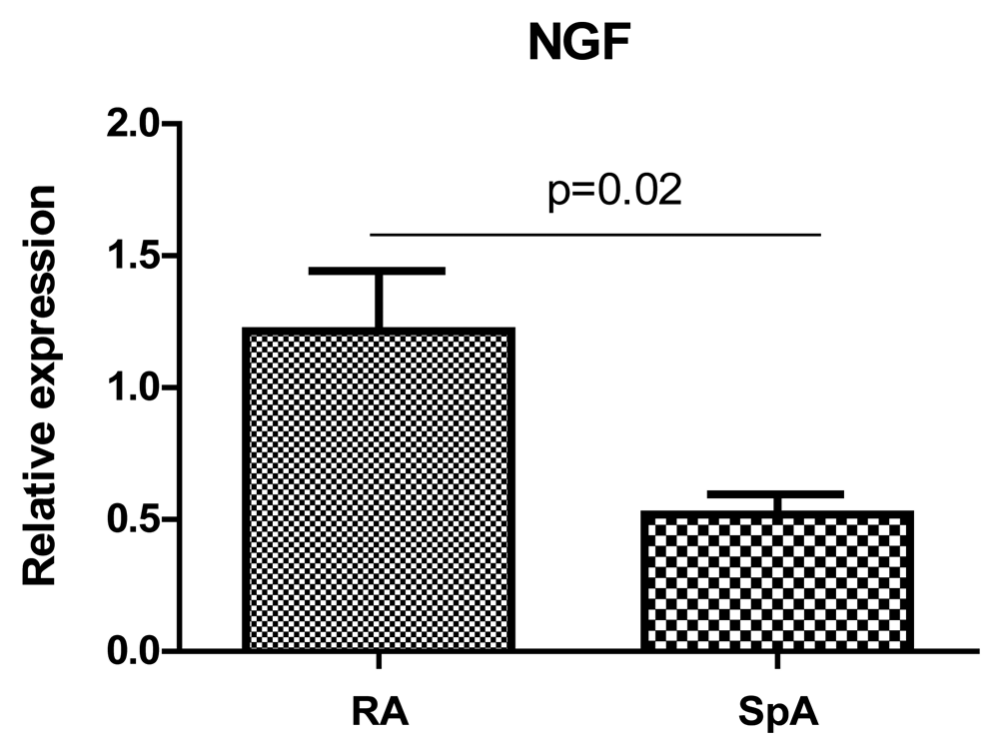
mRNA expression of NGF in synovial tissue samples. mRNA expression of nerve growth factor (NGF) as the prototype of neurotrophins was measured in the synovium of both 10 spondyloarthritis (SpA) and 10 rheumatoid arthritis (RA) patients; the expression levels were compared with each other (relative expression) showing a twice as high and thus significantly higher NGF expression in RA as compared with SpA (*P *= 0.02).

As for the transcripts encoding the ligands, BDNF revealed high expression levels in all three groups (RA median: 163, SpA median: 92 with a high range from 71 to 444, OA median: 137). The highest mRNA expression in SF samples was found for NGF revealing significantly higher levels in RA and SpA (median 418 and 323, respectively) as compared with OA (median 49; *P *= 0.001 for both comparisons). Transcripts encoding NT-3 and NT-4 were expressed on lower levels and slightly higher by trend in RA as compared with SpA and OA. The NGF mRNA expression levels as determined in ST biopsies were found to be significantly higher in RA (mean expression level 1.6 ± 1.2 SD) as compared with SpA (mean expression level 0.7 ± 0.3 SD) patients (*P *= 0.02) indicating that NGF is produced locally, at least in the arthritic synovium of RA patients (Figure 3).

In agreement with our previous immunohistochemistry data on ST samples [7], the mRNA transcripts encoding both the high-affinity NGF receptor trkA and the common low-affinity receptor p75 revealed the highest expression levels in SF of the SpA and the RA group being significantly higher expressed as compared with the OA group. The highest values were found for trkA in the SpA group (trkA median in SpA 20 vs OA 4.7; *P *= 0.003; p75 median in SpA 13.4 vs OA 4.8; *P *= 0.03) and for p75 in the RA group (trkA median values: RA 16 vs OA 4.7; *P *= 0.004; p75 median values: RA25 vs OA 4.8; *P *= 0.01 as determined by the Mann Whitney U test). Expression levels of trkB and trkC receptors were clearly lower than the ones of trkA and p75. Expression of trkB and trkC in SpA and OA was similar. However, trkB expression in the RA group was significantly higher as compared with the OA group (trkB median in RA 7.7 vs OA 4.1; *P *= 0.04).

**Figure 4:**
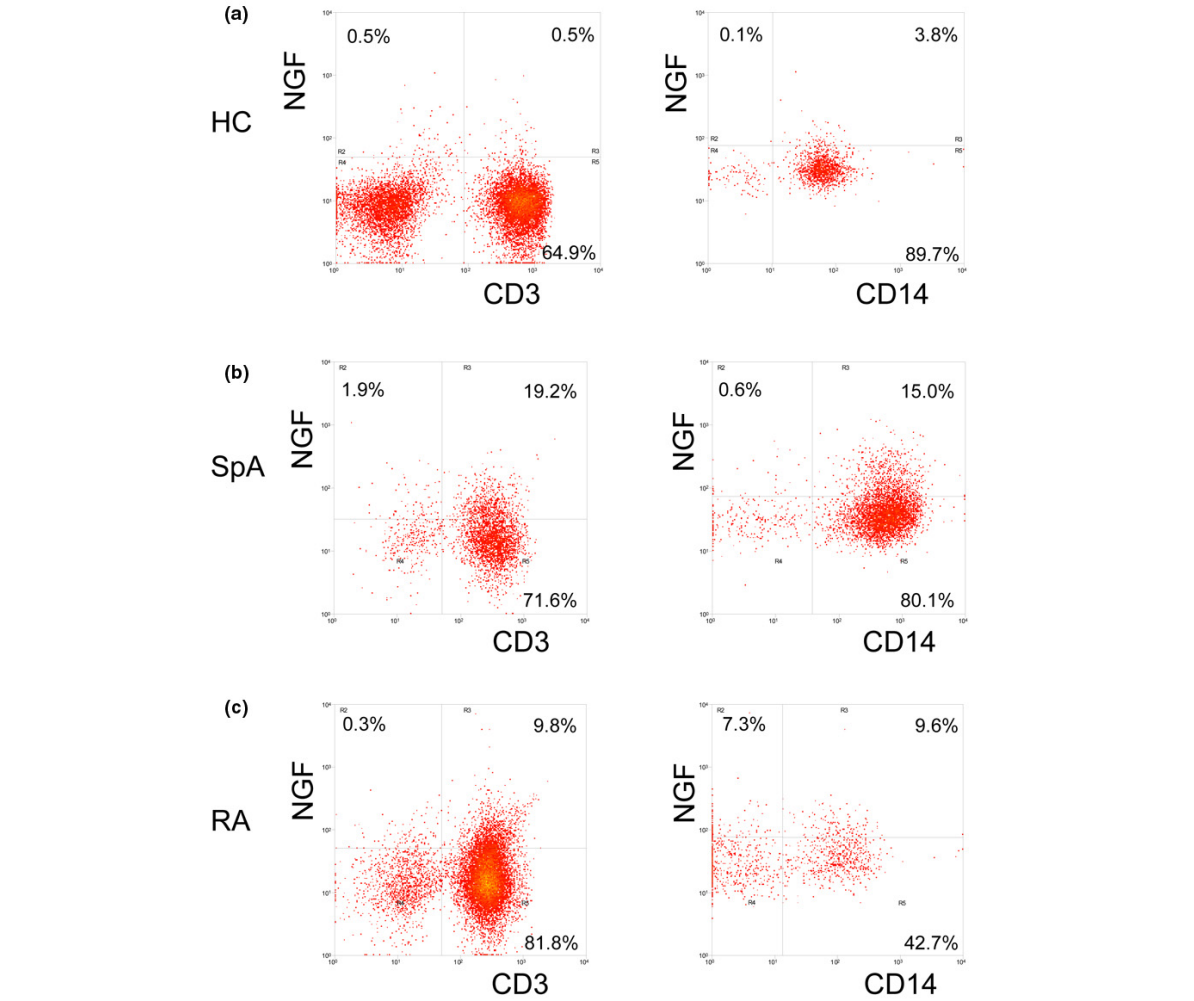
NGF staining by flow cytometry. PBMC of **(a) **healthy controls (HC) and **(b) **synovial fluid mononuclear cells (SFMC) from spondyloarthritis (SpA), and **(c) **rheumatoid arthritis (RA) patients were first stained with surface markers (CD3 and CD14) and permeabilized in order to enable intracellular detection of nerve growth factor (NGF). The cells were analysed by flow cytometry after setting lymphocyte (left column) and monocyte (right column) gates according to forward scatter vs side scatter properties of the cells. Dot plots of one representative individual of each group are shown.

We also measured NGF expression by staining on a single cell level using flow cytometry. Concomitant staining of cell surface markers and intracellular NGF revealed the presence of NGF in T lymphocytes (CD3+) and monocytes (CD14+). In contrast, B lymphocytes (CD19+) and nearly all natural killer cells (CD16+) were NGF negative in patients as well as in healthy controls. In both the SpA and the RA group, percentages of NGF expressing T lymphocytes and monocytes were considerably higher as compared with healthy controls (Figure 4). As we did notice clear mRNA expression for NGF not only in SF but also in ST, we additionally investigated the production of NGF by FLS. *In vitro *cultured FLS from RA as well as SpA did not secrete detectable levels of NGF, even upon stimulation with various proinflammatory cytokines (data not shown). Taken together, these data suggest that infiltrating T lymphocytes and myeloid cells are the main source of NGF in the inflamed peripheral joint.

## Discussion

The present study is a descriptive comprehensive quantitative expression analysis of mRNA transcripts encoding the four known human neurotrophins and their four corresponding receptors in the synovial compartment of arthritis patients.

The presence of neurotrophic factors in the inflamed joint has been described earlier [2,3]. Focusing on the NGF/trkA-p75 axis in our own and other work, we could previously demonstrate high trkA and p75 NGF receptor expression at the protein level in the inflamed ST in peripheral SpA synovitis. This expression was correlated with inflammatory disease activity and was downregulated by TNF-blocking treatment indicating that their expression is not constitutive but actively modulated in inflammation [7]. However, the high-affinity receptors trkB and trkC as well as the ligands NGF, NT-3, and NT-4 were expressed in the minority of patients or not detectable by immunohistochemistry.

In order to investigate the NGF/trkA-p75 axis as well as all other neurotrophic ligands and receptors at the transcript level, we used quantitative real-time RT-PCR to determine their expression in a larger panel of SpA and RA patients with active peripheral synovitis of the knee. Our data confirm the high expression of both the trkA and p75 NGF receptors at the transcript level in the synovial compartment of SpA and RA patients. Of note, we now provide evidence that the NGF ligand is also expressed in the SF and tissue biopsy samples of peripheral synovitis indicating that this system is active in chronic inflammatory arthritis. However, we need to state, that the high NGF transcript expression is in contrast to our previous ELISA data in SF samples [7]. This discrepancy between mRNA and protein expression has been reported earlier in studies on brain tissue [19]. Reasons for this phenomenon might involve post-transcriptional modifications of NGF [1,20]. We also can not exclude technical reasons such as the NGF antibody used for the previous quantitative immunoassay.

The cellular source of NGF in humans has been investigated in several studies. Under unstimulated conditions, NGF is produced mainly by CD4+ T and B lymphocytes [1,21]. Under inflammatory conditions such as allergy and arthritis, NGF can be produced, stored, and released by eosinophils, mast cells, lymphocytes, and synovial fibroblasts, as well as monocytes and macrophages [22]. Using intracellular FACS analysis, we could demonstrate the presence of the NGF protein in the two inflammatory arthritis groups on both CD3+ and CD14+ cells, that is, T lymphocytes and monocytes/macrophages, which are known to be involved in the major pathways of both SpA and RA. However, ST-derived FLS do not seem to produce NGF as measured by ELISA. This finding might indicate the mere pro-inflammatory potential of NGF as opposed to factors released by fibroblasts, which are predominantly involved in structural damage. On the other hand, we can not definitely rule out the production of NGF by FLS. One explanation would be, that FLS loose their ability to produce NGF when cultured over three passages *in vitro*. Another explanation would be that NGF is produced but not secreted, at least not in large amounts. Nevertheless, FLS are most likely one of the targets of NGF.

To date, the functional role of neurotrophins in inflammatory joint disorders is unclear. A pathogenic role for the NGF/trkA-p75 axis and other neurotrophins has been postulated for airway inflammation [23], atopic dermatitis [24], psoriasis [25], inflammatory bowel disease [26], and arthritis [2-7]. In inflammatory syndromes, NGF has been attributed to upregulating TNF-alpha, promoting the differentiation of B cells to plasma cells, enhancing chemotaxis and production of superoxide by neutrophils [22]. NGF is also involved in humoral immune responses by acting as an autocrine survival factor maintaining the viability of memory B-cells and macrophages [27]. NGF and its receptors have also tissue remodeling capacities exerting a strong fibrotic stimulus on skin and lung fibroblasts [28]. Upon binding to trkA, NGF induces its auto-phosphorylation and subsequently the activation of both phospholipase PLCγ and protein kinase C, which in turn activates the mitogen-activated protein kinase pathway involving the c-jun N-terminal, the p38, and the extracellular-regulated protein kinases (ERK1/2) all of which have been identified in arthritis as well. Interestingly, the wnt proteins, which have been described as regulating neurotrophin expression [29], have recently also been found to play a pathogenic role in spondyloarthritis [30]. In addition, NGF has been identified as a proangiogenic factor, another significant pathogenic pathway in chronic inflammatory arthritis such as SpA and RA [31,32].

## Conclusions

Taken together, this comprehensive analysis demonstrates the expression of the pleiotropic NGF and its two receptors in peripheral synovitis of SpA and RA. The knowledge of neurotrophin expression on cells from the inflamed synovial compartment in arthritis patients adds to the potential evaluation of pathogenic mechanisms and the development of new therapeutic strategies (e.g. the pharmacological blockade of the NGF receptor and their signaling pathway by using receptor antagonists). Our findings prompt further functional as well as clinical studies on the role of neurotrophins and their therapeutic potential in arthritis.

## Abbreviations

BDNF: brain-derived growth factor; BSA: bovine serum albumin; Ct: cycle threshold; DMEM: Dulbecco's modified eagle's medium; ELISA: enzyme-linked immunosorbent assay; FACS: fluorescence-activated cell sorter; FCS: fetal calf serum; FLS: fibroblast-like synoviocytes; G3PDH: glyceraldehyde 3-phosphate dehydrogenase; mAb: monoclonal antibody; NGF: nerve growth factor; NSAID: non-steroidal anti-inflammatory drug; NT: neurotrophin; OA: osteoarthritis; PBMC: peripheral blood mononuclear cells; PBS: phosphate-buffered saline; RA: rheumatoid arthritis; RT-PCR: reverse transcription polymerase chain reaction; SD: standard deviation; SpA: spondyloarthritis; SF: synovial fluid; SFMC: synovial fluid mononuclear cells; ST: synovial tissue; TNF: tumor necrosis factor; trk: tyrosine kinase.

## Competing interests

The authors declare that they have no competing interests.

## Authors' contributions

CB performed the RT-PCR experiments, the analysis of the data, and drafted the manuscript. NY did the FLS isolation and the ELISA. MB provided technical assistance in collecting the samples. RJ performed the FACS analysis. RES, HZ, PPT, and DB provided assistance in interpretation of the data and drafting the manuscript. MR designed the study and provided assistance in analysis and interpretation of the data and drafting the manuscript.

## References

[B1] Tessarollo L (1998). Pleiotropic functions of neurotrophins in development. Cytokine Growth Factor Rev.

[B2] Aloe L, Tuveri MA, Carcassi U, Levi-Montalcini R (1992). Nerve growth factor in the synovial fluid of patients with chronic arthritis. Arthritis Rheum.

[B3] Pozza M, Guerra M, Manzini E, Calza L (2000). A histochemical study of the rheumatoid synovium: focus on nitric oxide, nerve growth factor high affinity receptor, and innervation. J Rheumatol.

[B4] Wu Z, Nagata K, Iijima T (2000). Immunohistochemical study of NGF and its receptors in the synovial membrane of the ankle joint of adjuvant-induced arthritic rats. Histochem Cell Biol.

[B5] Iannone F, De Bari C, Dell'Accio F, Covelli M, Patella V, Lo Bianco G, Lapadula G (2002). Increased expression of nerve growth factor (NGF) and high affinity NGF receptor (p140 TrkA) in human osteoarthritic chondrocytes. Rheumatology (Oxford).

[B6] Grimsholm O, Guo Y, Ny T, Forsgren S (2008). Expression patterns of neurotrophins and neurotrophin receptors in articular chondrocytes and inflammatory infiltrates in knee joint arthritis. Cells Tissues Organs.

[B7] Rihl M, Kruithof E, Barthel C, De Keyser F, Veys EM, Zeidler H, Yu DT, Kuipers JG, Baeten D (2005). Involvement of neurotrophins and their receptors in spondyloarthritis synovitis: relation to inflammation and response to treatment. Ann Rheum Dis.

[B8] Dougados M, Linden S van der, Juhlin R, Huitfeldt B, Amor B, Calin A, Cats A, Dijkmans B, Olivieri I, Pasero G (1991). The European Spondylarthropathy Study Group preliminary criteria for the classification of spondylarthropathy. Arthritis Rheum.

[B9] Arnett FC, Edworthy SM, Bloch DA, McShane DJ, Fries JF, Cooper NS, Healey LA, Kaplan SR, Liang MH, Luthra HS (1988). The American Rheumatism Association 1987 Revised Criteria for the Classification of Rheumatoid Arthiritis. Arthritis Rheum.

[B10] Kellgren JH, Lawrence JS (1957). Radiological assessment of osteoarthritis. Ann Rheum Dis.

[B11] Baeten D, Bosch F Van den, Elewaut D, Stuer A, Veys EM, De Keyser F (1999). Needle arthroscopy of the knee with synovial biopsy sampling: technical experience in 150 patients. Clin Rheumatol.

[B12] Gu J, Rihl M, Märker-Hermann E, Baeten D, Kuipers JG, Song YW, Maksymowych WP, Burgos-Vargas R, Veys EM, De Keyser F, Deister H, Xiong M, Huang F, Tsai WC, Yu DT (2002). Clues to pathogenesis of spondyloarthropathy derived from synovial fluid mononuclear cell gene expression profiles. J Rheumatol.

[B13] Rihl M, Baeten D, Seta N, Gu J, De Keyser F, Veys EM, Kuipers JG, Zeidler H, Yu DT (2004). Technical validation of cDNA based microarray as screening technique to identify candidate genes in synovial tissue biopsy specimens from patients with spondyloarthropathy. Ann Rheum Dis.

[B14] Wendt K, Wilk E, Buyny S, Buer J, Schmidt RE, Jacobs R (2006). Gene and protein characteristics reflect functional diversity of CD56dim and CD56bright NK cells. J Leukoc Biol.

[B15] Bustin SA (2000). Absolute quantification of mRNA using real-time reverse transcription polymerase chain reaction assays. J Mol Endocrinol.

[B16] Pfaffl MW (2001). A new mathematical model for relative quantification in realtime RT-PCR. Nucleic Acids Res.

[B17] Fleige S, Walf V, Huch S, Prgomet C, Sehm J, Pfaffl MW (2006). Comparison of relative mRNA quantification models and the impact of RNA integrity in quantitative real-time RT-PCR. Biotechnol Lett.

[B18] Vandooren B, Cantaert T, ter Borg M, Noordenbos T, Kuhlman R, Gerlag D, Bongartz T, Reedquist K, Tak PP, Baeten D (2008). Tumor necrosis factor alpha drives cadherin 11 expression in rheumatoid inflammation. Arthritis Rheum.

[B19] Zhang HT, Li LY, Zou XL, Song XB, Hu YL, Feng ZT, Wang TT (2007). Immunohistochemical distribution of NGF, BDNF, NT-3, and NT-4 in adult rhesus monkey brains. J Histochem Cytochem.

[B20] Freund-Michel V, Frossard N (2008). The nerve growth factor and its receptors in airway inflammatory diseases. Pharmacol Ther.

[B21] Lambiase A, Bracci-Laudiero L, Bonini S, Bonini S, Starace G, D'Elios MM, De Carli M, Aloe L (1997). Human CD4+ T cell clones produce and release nerve growth factor and express high-affinity nerve growth factor receptors. J Allergy Clin Immunol.

[B22] Bonini S, Rasi G, Bracci-Laudiero ML, Procoli A, Aloe L (2003). Nerve growth factor: neurotrophin or cytokine?. Int Arch Allergy Immunol.

[B23] Rochlitzer S, Nassenstein C, Braun A (2006). The contribution of neurotrophins to the pathogenesis of allergic asthma. Biochem Soc Trans.

[B24] Dou YC, Hagstromer L, Emtestam L, Johansson O (2006). Increased nerve growth factor and its receptors in atopic dermatitis: an immunohistochemical study. Arch Dermatol Res.

[B25] Raychaudhuri SP, Raychaudhuri SK (2004). Role of NGF and neurogenic inflammation in the pathogenesis of psoriasis. Prog Brain Res.

[B26] Reinshagen M, von Boyen G, Adler G, Steinkamp M (2002). Role of neurotrophins in inflammation of the gut. Curr Opin Investig Drugs.

[B27] Manni L, Lundeberg T, Fiorito S, Bonini S, Vigneti E, Aloe L (2003). Nerve growth factor release by human synovial fibroblasts prior to and following exposure to tumor necrosis factor-alpha, interleukin-1 beta and cholecystokinin-8: the possible role of NGF in the inflammatory response. Clin Exp Rheumatol.

[B28] Micera A, Vigneti E, Pickholtz D, Reich R, Pappo O, Bonini S, Maquart FX, Aloe L, Levi-Schaffer F (2001). Nerve growth factor displays stimulatory effects on human skin and lung fibroblasts, demonstrating a direct role for this factor in tissue repair. Proc Natl Acad Sci USA.

[B29] Patapoutian A, Backus C, Kispert A, Reichardt LF (1999). Regulation of neurotrophin-3 expression by epithelial-mesenchymal interactions: the role of Wnt factors. Science.

[B30] Diarra D, Stolina M, Polzer K, Zwerina J, Ominsky MS, Dwyer D, Korb A, Smolen J, Hoffmann M, Scheinecker C, Heide D van der, Landewe R, Lacey D, Richards WG, Schett G (2007). Dickkopf-1 is a master regulator of joint remodeling. Nat Med.

[B31] Lazarovici P, Marcinkiewicz C, Lelkes PI (2006). Cross talk between the cardiovascular and nervous systems: neurotrophic effects of vascular endothelial growth factor (VEGF) and angiogenic effects of nerve growth factor (NGF)-implications in drug development. Curr Pharm Des.

[B32] Szekanecz Z, Koch AE (2007). Mechanisms of disease: angiogenesis in inflammatory diseases. Nat Clin Pract Rheumatol.

